# Thermogelling 3D Systems towards Stem Cell-Based Tissue Regeneration Therapies

**DOI:** 10.3390/molecules23030553

**Published:** 2018-03-02

**Authors:** Xiaoyuan Wang, David James Young, Yun-Long Wu, Xian Jun Loh

**Affiliations:** 1 Fujian Provincial Key Laboratory of Innovative Drug Target Research, School of Pharmaceutical Sciences, Xiamen University, Xiamen 361102, China; 32320151154228@stu.xmu.edu.cn; 2 Faculty of Science, Health, Education and Engineering, University of the Sunshine Coast, Maroochydore 4558, Queensland, Australia; dyoung1@usc.edu.au; 3 A*STAR (Agency for Science, Technology and Research), Institute of Materials Science and Engineering, 2 Fusionopolis Way, Innovis, #08-03, Singapore 138634, Singapore

**Keywords:** thermogel, 3D culture, stem cell, tissue engineering

## Abstract

Stem cell culturing and differentiation is a very important research direction for tissue engineering. Thermogels are well suited for encapsulating cells because of their non-biotoxic nature and mild sol-gel transition as temperature increases. In particular, thermogels provide a 3D growth environment for stem cell growth, which is more similar to the extracellular matrix than flat substrates, so thermogels as a medium can overcome many of the cell abnormalities caused by 2D cell growth. In this review, we summarize the applications of thermogels in cell and stem cell culture in recent years. We also elaborate on the methods to induce stem cell differentiation by using thermogel-based 3D scaffolds. In particular, thermogels, encapsulating specific differentiation-inducing factor and having specific structures and moduli, can induce the differentiation into the desired tissue cells. Three dimensional thermogel scaffolds that control the growth and differentiation of cells will undoubtedly have a bright future in regenerative medicine.

## 1. Introduction

Tissue engineering is a promising approach for treating damaged tissues or organs, without the need for donations from other sources. The most widely studied technique in this respect involves the use of a patient’s own stem cells to grow functional cells to replace the damaged tissue. Stem cell growth and differentiation, however, requires an advanced biomimetic extracellular matrix (ECM) that can maintain structure in three dimensions [1,2]. It is, of course, easier to grow cells in a two dimensional environment, for example, in a culture dish made of polystyrene. However, cellular growth in this unnatural shape can lead to unexpected phenotype changes and abnormal biomarker expressions. Human breast epithelial cells, for example, become tumor-like when they are cultured in 2D conditions but return to their original status upon culturing in a three-dimensional environment [3]. Chondrocytes become fibroblast-like in a 2D culture due to elevated type I collagen expression, while more type II collagen expression is observed in a 3D culture [4]. It would appear, therefore, that cells cultured in a 3D scaffold is closer to a normal growth pattern. Understanding and guiding 3D cell culture is a key issue for tissue regeneration. Hydrogels are emerging as one of the most promising materials for the preparation of an artificial ECM [5] In contrast to natural systems, synthetic hydrogels are easier to control with respect to composition, conformation and degradability. More importantly, synthetic hydrogels are reproducible in manufacturing, and they are now being investigated for large scale cell culturing and differentiation control [6,7,8].

Thermogels are well suited for encapsulating cells because of their mild sol-gel transition as temperature increases [9,10]. Ideally, stem cells can be encapsulated in a non-biotoxic thermogel, which is liquid at a lower temperature and then is physically crosslinked in a more solid state when the temperature increases to body temperature. It is worth mentioning that the sol-gel transition process is mild and harmless for cells in contrast to chemical or photochemical crosslinking systems [8]. Furthermore, as an added advantage for future body repair, the 3D scaffold thermogel can be injected at the target site using a conventional syringe, without the need for complicated surgery [11]. A thermogel matrix can fit any shape, even in irregular organ locations, and can encapsulate a cocktail of differentiating and growth factors. Moreover, hydrogels can provide some protection to cells from damaging free radicals, ultraviolet radiation and reactive chemicals, which might inhibit cell viability, proliferation or differentiation [6].

### The Early Studies of Thermogel 3D Systems in Culture Cell

**Figure 1:**
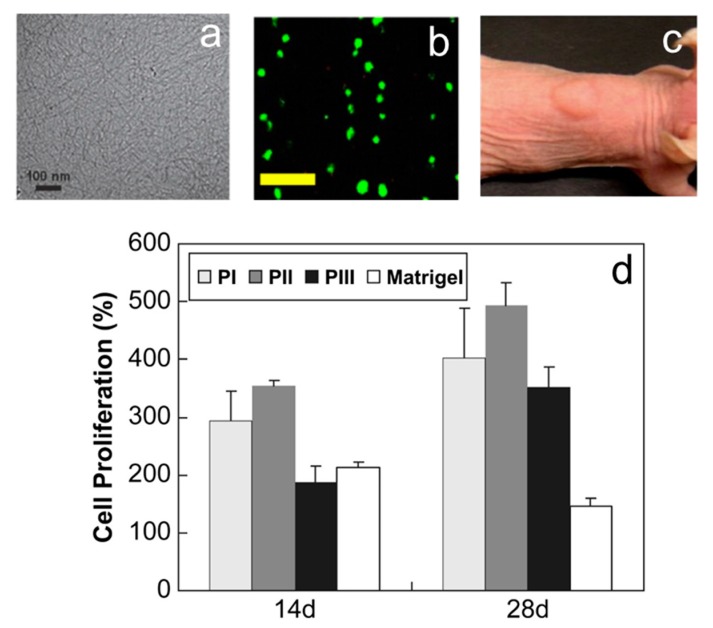
(**a**) Nanostructure of L/D-PA-PLX-L/D-PA. The scale = 100 nm; (**b**) Cell morphology in 28th day in L/D-PA-PLX-L/D-PA cultured systems. The scale bar is 200 μm; (**c**) The formation of thermogels after the nude mice subcutaneous injection of the PII thermogel; (**d**) Chondrocyte proliferation rates within a thermogel 3D environment. (reproduced with permission from [4]).

Thermogels have been widely studied in the past two decades for their biomedical applications, including for drug delivery [12,13,14], tissue engineering [15,16,17], post-surgical adhesion prevention [18,19], embolization and wound dressing [20,21]. Recently, researchers have also focused on controlling the growth and differentiation of stem cells by adding various inducing factors for growth and differentiation into the thermogel matrix, with some success. A study [4] have reported a thermogel made of PA-PLX-PA block copolymer (Figure 1) that could encapsulate chondrocytes at low temperature, and form a 3D scaffold containing these cells at 37 °C. Copolymer L/D-PA-PLX-L/D-PA (PII) proved to be an outstanding 3D culture substrate for the growth and proliferation of chondrocytes, maintaining their spherical phenotype (Figure 1b).

**Figure 2:**
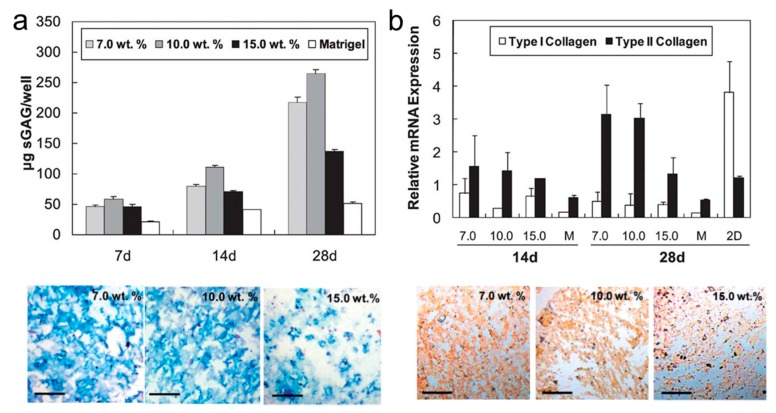
(**a**) sGAG and alcian blue stained image indicates chondrocytes in thermogels at different initial concentration of PA-PLX-PA. The scale bar represents 200 μm; (**b**) Culturing chondrocytes in different 2D or 3D culture medium, the mRNA expression of type I and type II collagen was analyzed at the 14th and 28th day. M (Matrigel^MT^) and 2D cultured system were also used for comparison. Type II collagen immunostaining image of chondrocytes at the 28th day. The scale bar is 200 μm (reproduced with permission from [22]).

This group also investigated the effect of block copolymer concentration and hydrogel structure on the behavior of chondrocyte culturing [22]. The viability of chondrocytes was excellent in 7.0–10.0 wt.% PA-PLX-PA gel, with elevated type II collagen and sGAG expression (Figure 2a,b), indicating the importance of micromechanical cues such as the hydrogel’s moduli and nanofiber thickness for 3D cell growth.

**Figure 3:**
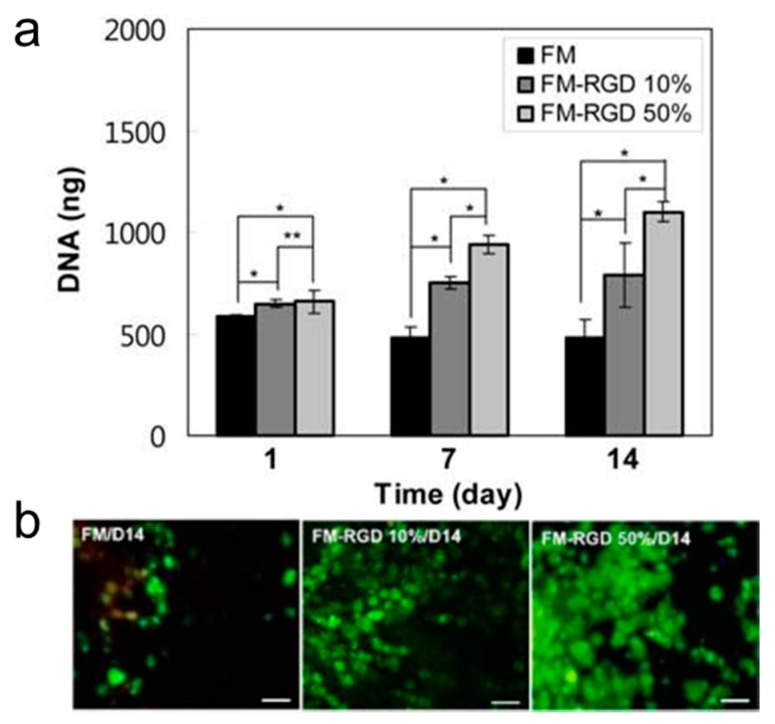
(**a**) Comparison of gene expression in thermogels of different RGD content, * and ** indicate *p* < 0.01 and *p* > 0.01 by the Student *t*-test; (**b**) Confocal microscopy images of cells in different RGD content thermogels at 14 day. The scale bar is 200 μm (reproduced with permission from [23]).

The Han and Jeong groups [23] have reported the utilization of a thermogel made of Arg-Gly-Asp (RGD) modified pluronic F127 dimethacrylate (FM-RGD) polymer, as a three dimensional cell culture scaffold. This FM-RGD thermogel exhibited a modulus of 8900 Pa at a culture temperature of 37 °C. FM-RGD showed significant improvement in cell viability, cell proliferation, gene expression and longer maintenance time for spherical phenotypes of chondrocytes than did the corresponding F127 dimethacrylate (FM) thermogel without RGD. Increasing RGD content from 10% to 50% led to chondrocyte proliferation and gene expression improvements (Figure 3a,b). This study highlighted the importance of a thermogel’s chemical composition in the design of an artificial 3D ECM.

## 2. Hydrogel-Based 3D Culturing and Differentiation of Stem Cells

Stem cells with differentiation ability are considered the next generation of therapeutic tools [24,25]. Embryonic stem cells (ESC), induced pluripotent stem cells (iPSC) and mesenchymal stem cells (MSCs) are commonly used in stem cells research. Although ESC and iPSC have potentials to differentiate into various kinds of cells, the application of ESC is still subject to ethical issues in certain parts of the world. Mesenchymal stem cells (MSCs), a kind of adult stem cells, can serve as a better candidate for research since it can also differentiate into other cells and can avoid the shortcomings of ESC and iPSC. Mesenchymal stem cells (MSCs) have important characteristics of immunosuppression, self-renewal and differentiation into mesenchymal cell lines, such as bone, cartilage, adipose tissue, neurons, or muscles [26,27]. Typical mesenchymal stem cell phenotypes include bone marrow-derived MSCs (BMSCs), tonsil tissue-derived MSCs (TMSCs) and adipose tissue-derived stem cells (ADSCs).

**Scheme 1:**
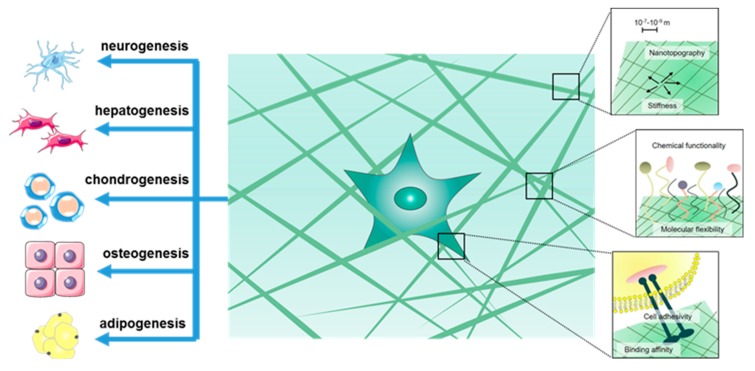
Hydrogel-based 3D culture scaffold for stem cell culturing and differentiation.

**Figure 4:**
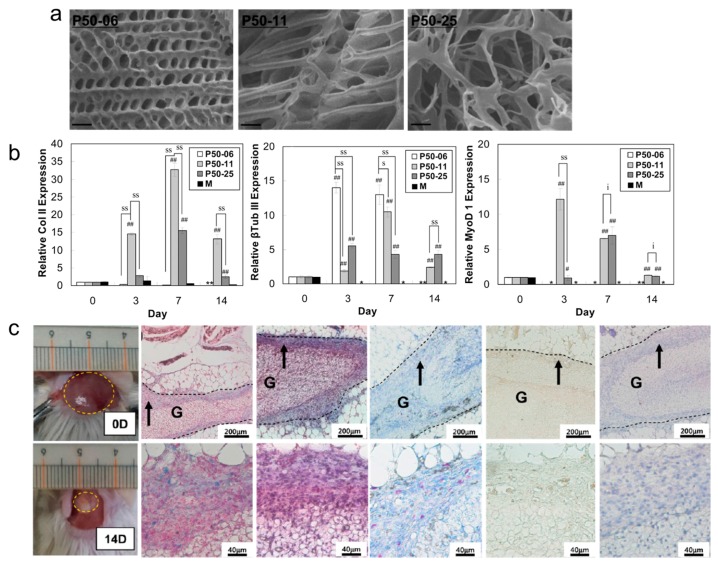
(**a**) SEM images of three kinds of PEG-L-PA thermogels prepared at 37 °C. The scale bar is 10 μm; (**b**) In vitro studies, Col II, βTub III, and MyoD1 gene expressions of ADSCs in PEG-L-PA thermogels, # and ## indicate *p* < 0.05 and *p* < 0.01 by Student *t*-test; s, ss, and i indicate *p* < 0.05, *p* < 0.01, and *p* > 0.05 by Student *t*-test; (**c**) In vivo mouse model of injected thermogel in situ with histological staining by alcian blue, Masson’s trichrome, toluidine blue, alizarin red S, and oil red O around the implant at 14 days. The regions marked by arrows are enlarged. The thermogel regions (G) are specified by the dotted curve. The scale bars are 200 and 40 μm, respectively (reproduced with permission from [10]).

Three dimensional culture matrices provide oxygen and nutrients to stem cells, impact the alignment of the cytoskeleton and affect cell morphology, gene expression and protein production [28]. In the past ten years, extensive research has been devoted to understanding the factors which regulates MSC differentiation into specific cells and tissues (Scheme 1). The chemical functional groups, hardness or topography of the substrate, the size of the niche, and the geometry of the patterned surface can affect stem cell differentiation. A recent study [10] have developed an injectable poly(ethylene glycol)-b-poly(l-alanine) (PEG-L-PA)s block copolymer that can be used to culture adipose tissue-derived stem cells (ADSCs). These PEG-L-PAs were composed of PEG with molecular weight of 5000 Da and L-PA with molecular weight of 620 Da, 1100 Da, or 2480 Da (Figure 4a). ADSCs could maintain spherical geometry within this hydrogel.

The effects of biomarkers lipoprotein lipase (LPL), type III β-tubulin (βTub III), osteocalcin (OCN) type II collagen (Col II), and myogenic differentiation factor 1 (MyoD1) were studied for stem cell differentiation into fat, neuronal, bone and other tissue cells. In vitro studies of cells embedded in PEG-L-PA hydrogel indicated high expression of Col II and moderate expression of βTub III and MyoD 1 (Figure 4b), potentially developing ADSCs into chondrocytes. In vivo studies also showed that ADSCs could undergo chondrogenic differentiation (Figure 4c). However, lipogenesis (LPL) and osteogenesis (OCN) biomarkers were both undetectable in vitro and in vivo.

In another study, Jeong’s group developed poly(ethylene glycol)-poly(l-alanine-co-l-phenyl alanine) (PEG-PAF) thermogels for three dimensional TMSC culturing [29]. This thermogel could serve as a support to encapsulate TMSCs together with various growth factors. The in vitro and in vivo results both indicated that type II collagen and sulfated glycosaminoglycan were highly expressed in TMSCs incorporated in to PEG-PAF thermogel, indicating the preferential differentiation of TMSCs towards chondrogenesis. In this context, it can be concluded that cytoskeleton—matrix adhesion is an important trigger for stem cell differentiation.

**Figure 5:**
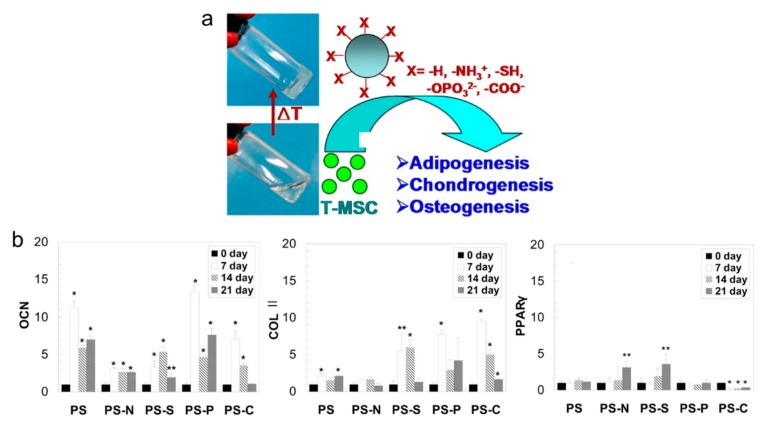
(**a**) Schematic of controlling specific differentiation of TMSCs by adding surface-modified microspheres in PEG-L-PA thermogels; (**b**) OCN, Col II, PPARγ expression of TMSCs were analysis in 3D cultured thermogel, * and ** indicate *p* < 0.01 and *p* < 0.05 by Student *t*-test (reproduced with permission from [30]).

As an improvement to a previous design, the same group also demonstrated a thermogel made of PEG-L-PAs that incorporated polystyrene microspheres [30]. These microspheres were designed to be within the size range 100 to 800 µm with various functional groups. An mRNA expression experiment indicated that the TMSCs differentiated into adipocytes in the thermogels with ammonium or thiol modified microspheres; differentiated into chondrocytes in a thermogel with thiol-, phosphate, or carboxylate modified microspheres; and differentiated into osteoblasts in the thermogels with phosphate-, carboxylate-modified microspheres (Figure 5a,b). TMSCs could be manipulated to preferentially differentiate into a specific cell type by controlling the functional groups of the microspheres in the thermogel.

## 3. Stem Cell Specific Differentiation

Chemical groups, stiffness, topography of substrates and geometry of surfaces can be manipulated to direct stem cell differentiation [31,32,33,34]. Many studies have shown that adding appropriate differentiation factors can induce specific differentiation of stem cells, while the thermogels can be a good carrier for encapsulating differentiation factors and stem cells. On the other hand, another studies have also shown that the matrix stiffness can also induce stem cell specific differentiation, Soft thermogels, with brain-like stiffness, can induce stem cells to undergo neurogenic differentiation; stiffer matrices, with stiffness similar to liver, can induce stem cells into hepatogenic differentiation, and rigid thermogels can induce stem cell to undergo osteogenic differentiation. Moreover, it has been proposed that tethering and matrix porosity also can influence stem cell differentiation [35].

### 3.1. Scaffold Induced Neuronal Differentiation

**Figure 6:**
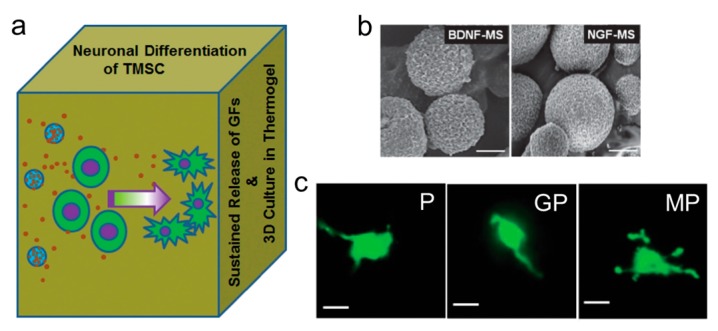
(**a**) PEG-L-PA thermogel as a 3D cell culture matrix incorporating TMSCs and neuronal growth factor loaded microspheres; (**b**) NGF-loaded and BDNF-loaded alginate microspheres. Scale bar is 1 μm; (**c**) Image of cells cultured in PEG-L-PA thermogel with different growth factors: P indicates the absence of growth factor, GP indicates thermogel incorporating growth factors, MP indicate thermogel incorporating growth factor-loaded microspheres. Scale bar is 20 μm (reproduced with permission from [38]).

Neurodegenerative injury or disease poses a serious threat to quality of life as we grow older and the ability of neural tissue for self-repair is limited [36,37]. A recent study [38] suggests that PEG-L-PA thermogel might facilitate TMSC neuroforming (Figure 6a). In this study, microspheres, with loading of neuronal growth factors (i.e., BDNF or NGF, shown in Figure 6b), were co-encapsulated at a temperature of 37 °C. 

These cells showed exuberant vitality in this 3D culture medium and grew a structure that was similar to nerve fibers after a period of cultivation (Figure 6c). The associated neurobiomarkers were highly expressed, suggesting that the cells could differentiate and form mature nerve cells.

### 3.2. Scaffold Induced Hepatogenic Differentiation

Liver disease is a leading cause of morbidity and mortality. Currently, the only effective treatment for acute liver failure and advanced liver disease is orthotopic liver transplantation [39,40]. Donor organ deficiencies, surgical complications, immune rejection and expense limit this treatment option. Cell transplantation is a potential alternative [41,42]. However, developing cell-based therapies for the treatment of liver diseases requires reliable and renewable sources of hepatocytes.

**Figure 7:**
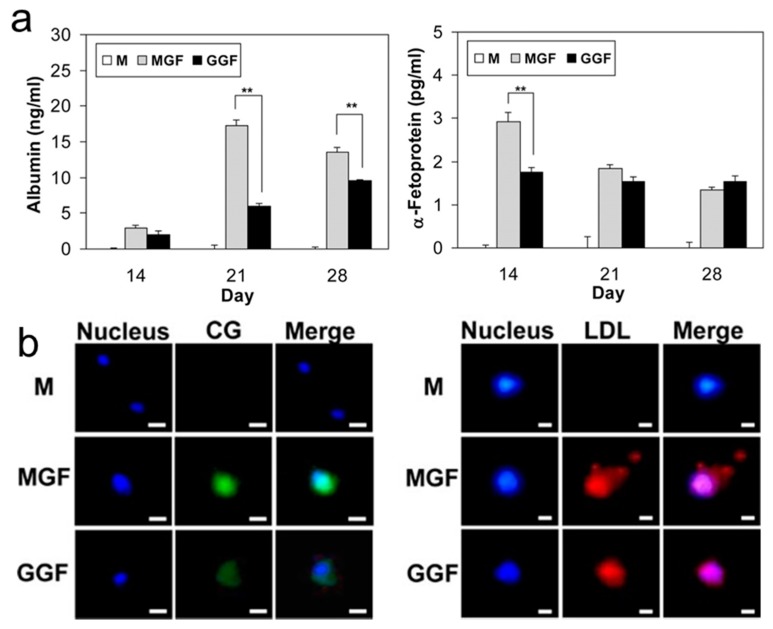
(**a**) Expression of albumin and α-fetoprotein by TMSCs in PEG-L-PA thermogel, * and ** indicate *p* < 0.05 by Student *t*-test; (**b**) Images of cellular uptake of CG and LDL in PEG-L-PA thermogels at 28 days. The scale bar is 10 μm (reproduced with permission from [43]).

A PEG-L-PA thermogel system has been developed for growing hepatocytes [43]. Importantly, the sol-gel in this system undergoes transition at 37 °C to provide a gel with a modulus of about 1000 Pa which is similar to acellular liver tissue. Hepatogenic growth factors present in the thermogel system change the morphology and aggregation of cells with expressions of hepatocyte-specific biomarkers and concomitant change of behavior of metabolic function. In the absence of hepatogenic growth factors, the production of hepatocyte biomarkers was slight and metabolic functions were weak. Production of albumin and α-fetoprotein were significant (Figure 7a) in both MGF (medium with hepatogenic growth factors) and GGF (3D matrix coencapsulated hepatogenic growth factors). The uptake of low-density lipoprotein (LDL) and cardiogreen (CG) was apparent in MGF and GGF (Figure 7b), which is typical metabolic function of hepatocytes. The conclusion can be drawn that the PEG-L-PA/TMSCs/growth factor system is a promising 3D scaffold for the differentiation of TMSCs into hepatocytes.

**Figure 8:**
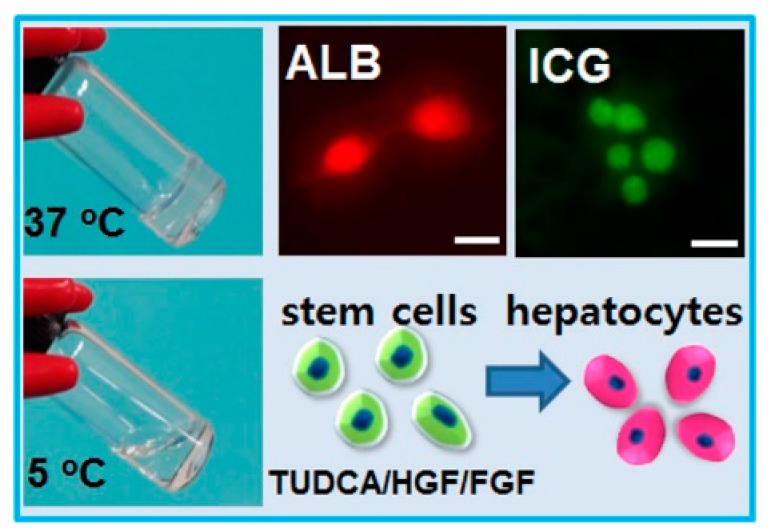
PEG-L-PA polypeptide thermogel system for hepatogenic differentiation of TMSCs. (reproduced with permission from [16]).

Some researchers [16] further investigated a PEG-L-PA thermogel system integrating TMSCs and hepatogenic differentiating factors. Hepatic biomarkers were expressed at both mRNA and protein levels and hepatic functionality such as CG and LDL uptake and albumin and urea production was observed (Figure 8).

### 3.3. Scaffold Induced Chondrogenesis Differentiation

**Figure 9:**
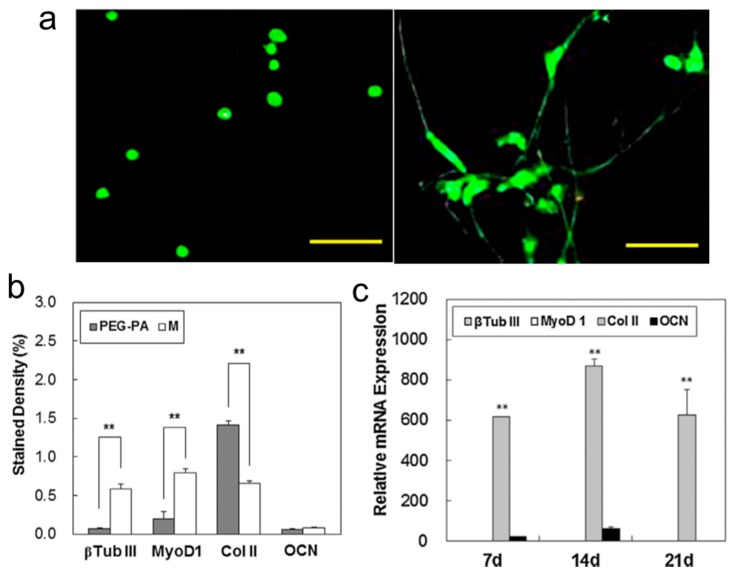
(**a**) Morphology of cells in the PEG-PA thermogel (left) and Matrigel^TM^ (right) at the 21st day. The scale bar is 40 μm; (**b**). Immunofluorescent assay using Image J software, ** indicates *p* < 0.05 Student *t*-test; (**c**) Gene expression in vivo of the BMSCs, ** indicates *p* < 0.05 by Student *t*-test (reproduced with permission from [44]).

Excellent viability was observed when bone-marrow-derived mesenchymal stem cells (BMSCs) were encapsulated into both PEG-PA thermogel and Matrigel™ (Figure 9a). These BMSCs underwent chondrogenesis and myogenesis in the PEG-PA thermogel (Figure 9b), whereas neurogenesis was observed in the Matrigel™. In addition, an in vivo study in mice showed the prime formation of Col II and sulfated glycosaminoglycan (Figure 9c), indicating that chondrogenic differentiation of the BMSCs dominated in the implanted PEG-PA gel [44].

**Figure 10:**
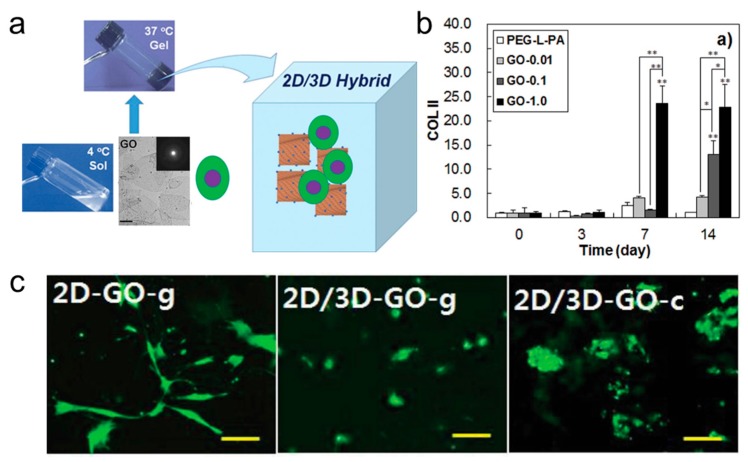
(**a**) Schematic of the 2D/3D thermogel system for culturing TMSCs; (**b**) Expression of COL II at different GO concentrations, * or ** on the bar graph indicates the significance of the 2D/3D hybrid system compared with the PEG-L-PA 3D culture system; (**c**) Cell morphology after 14 days of cell culture. Left: 2D culture with GO, middle: 3D culture with GO/PEG-L-PA, and right: 3D culture with GO/PEG-L-PA and enriched with TGF-β3. The scale bar is 100 μm (reproduced with permission from [45]).

Physicochemical parameters of structure such as topography, roughness of substrates, mechanical properties, and biochemical functional groups control stem cell differentiation. However, the dimensionality of the material, i.e., 1D fibers, 2D sheets, and 3D hydrogels also plays a role. The Jeong group have developed a 2D/3D hybrid cell culture sccaffold involving TMSCs suspended in thermogelling PEG-L-PA aqueous solution together with graphene oxide (GO) (Figure 10a) [45]. The spherical morphology of the cells was maintain in this 2D/3D culture scaffold of GO/PEG-L-PA. Cells extensively aggregated when TGF-β3 enriched chondrogenic culture media was used (Figure 10c), and expression of COL II, a chondrogenic biomarkers, increased significantly (Figure 10b).

### 3.4. Scaffold Induced Osteogenic Differentiation

**Figure 11:**
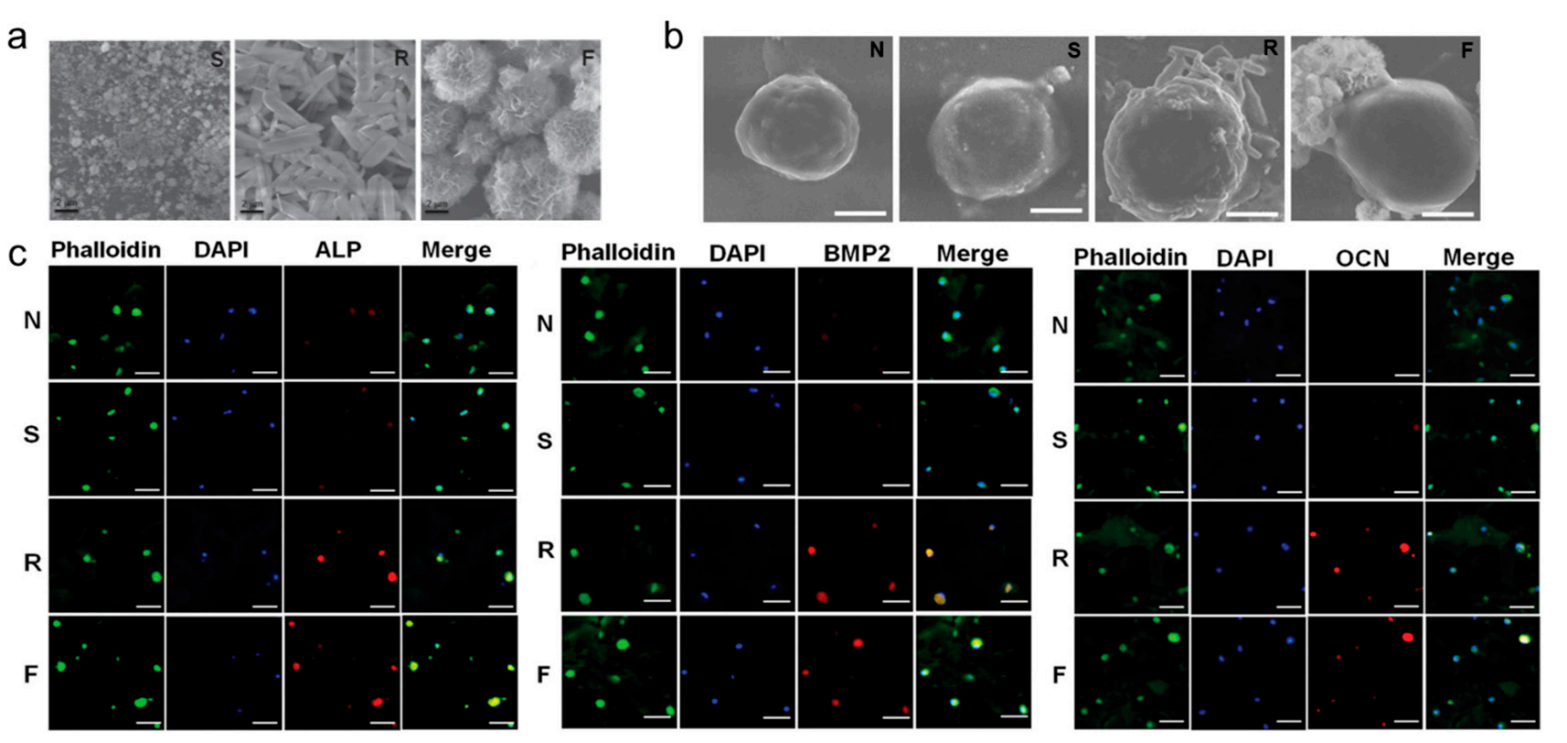
(**a**) SEM images of calcium phosphate crystals produced under different conditions. The scale bar is 2 μm; (**b**) SEM images of interactions between cells and cell culture matrix of thermogels. The scale bar is 5 μm; (**c**) Osteogenic biomarker production at protein level during the osteogenic differentiation of TMSCs. N represents the unmixed PEG-PAF. S represents the thermogel with calcium phosphate nanoparticles from Sigma-Aldrich. R represents the thermogel rod-like morphology calcium phosphate mesocrystals, F represents the thermogel flower-like morphology calcium phosphate mesocrystals (reproduced with permission from [49]).

Every year there are a large number of clinical bone transplants, and with the aging population, osteoporosis is becoming an increasingly serious disease [46]. Osteogenesis through stem cell differentiation is considered to be a promising alternative to bone transplantation [47,48]. Mesoscience is a key platform at the interface between biology and chemistry and encompasses the synthesis of precise mesostructures and characteristics for the cell-material interface. It is difficult for hydrogels themselves to function as a scaffold for osteogenesis due to the lack of a force transduction mechanism between the thermogel and cell. However, inorganic/organic mesocomposite materials can overcome this limitation of a single thermogel system for osteogenic differentiation. This technology involves mesocrystals embedded in the thermogel matrix to improve interactions between the cell and material leading to osteogenesis. One such system involves inorganic/organic complexes containing 4–8 μm calcium phosphate mesogens (Figure 11a) which proved more effective at osteogenic differentiation of TMSCs relative to nanoparticle incorporating systems or hydrogels alone [49]. Osteogenic biomarkers (Figure 11c) at the mRNA and protein levels were highly expressed. This composite system provides both a hard surface for binding cells/proteins and a mild scaffold for holding cells in place (Figure 11b).

### 3.5. Scaffold Induced Adipogenic Differentiation

**Figure 12:**
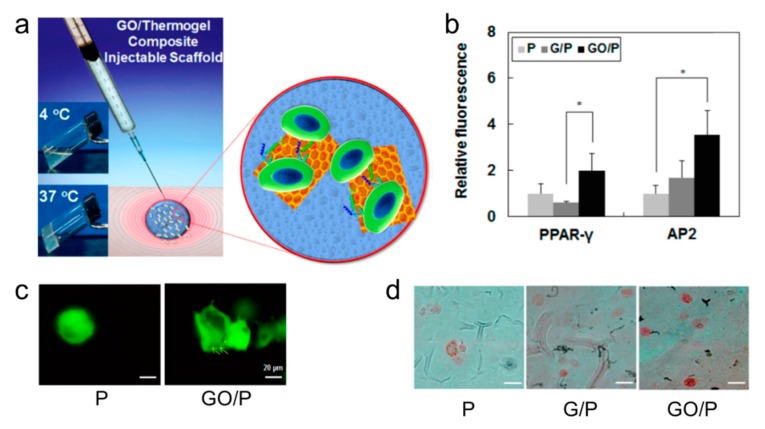
(**a**) Schematic presentation of the 3D culture of TMSCs in a GO/P composite injectable system; (**b**) Semiquantitative analysis of the fluorescence images, * indicates *p* < 0.05 by Student *t*-test; (**c**) Comparative images of a thermogel (P) system, and graphene oxide-incorporated thermogel (GO/P) system at day 14. The arrows in the GO/P system image indicate lipid vesicles. The scale bar is 20 μm; (**d**) Oil-red staining of the TMSCs incubating in the P, G/P, and GO/P systems respectively on the 14th day. The scale bar is 50 μm (reproduced with permission from [50]).

Adipogenic differentiation in a 3D culture scaffold can be used for reconstructive surgery of fat tissues [50]. Again, adipogenic differentiation was achieved in a 2D/3D culture scaffold by using TMSCs (Figure 12a). A graphene oxide/polypeptide thermogel (GO/P) system produced adipogenic biomarkers, such as PPAR-γ, AP2 at a significantly enhanced level compared to a pure thermogel system (Figure 12b). Moreover, when insulin, an adipogenic differentiation factor, was added in the GO/P thermogel, it preferentially adhered to GO to provide sustained differentiation (Figure 12c). However, in the presence of G, insulin denatured partially and obstructed adipogenic differentiation (Figure 12d).

In addition to thermogels for culturing stem cell, there are many studies which use other hydrogels to culture stem cells. These different hydrogels have their own advantages. For example, Lowman group try to use poly(*N*-isopropylacrylamide)-co-poly(ethylene glycol), an injectable hydrogel, to repair spinal cord injury [51] and the Lee group investigated an alginate hydrogel with RGD peptide for adipogenic differentiation of adipose-derived stromal cells [52]. Furthermore, the Hoyland group has studied a chitosan hydrogel, which could be used for the regeneration of intervertebral discs [53].

## 4. Conclusions and Perspectives

Adjusting the physical and chemical properties of a commonly used thermogel system provides a platform to manipulate stem cell differentiation by tuning substrate stiffness, porosity and ligand tethering. However, for practical applications of this scaffold in reconstructive surgery, efficiency of differentiation to adipose, hepatic and cartilage tissue needs to be improved. Thermogel composite systems are proving a promising tool, providing a minimally invasive injectable system, generating tissue volume and an excellent 3D matrix for differentiation of the incorporated stem cells [54,55].

Although 3D culture systems offer possible advantages relative to 2D culture systems, there are many constraints to be considered. Angiogenesis in these artificial 3D scaffolds, for example, is necessary to provide nutrients and remove waste [56]. Moreover, it is challenging to alter one property of a thermogel without disturbing other properties. Increasing stiffness, for example, will affect exchange of oxygen and nutrients in the thermogel. Effective control of stem cells will require scaffolds with multiple optimized properties including stiffness, biotic factors, biodegradablity and porosity. Although a number of highly engineered, complex scaffolds have been designed over the past few years, most of these biomaterials are not yet suitable for commercialization.

On the positive side, modern microscale technologies open new opportunities. Scaffolds can be prepared using 3D printing and electrospinning at microscale and even nanoscale sizes [56,57,58]. Spatial positioning of biomolecules and terrain of scaffolds can be accurately controlled. Three dimensional thermogel scaffolds that control the growth and differentiation of cells will undoubtedly have a bright future in regenerative medicine.

Due to the unpredictable nature of living systems and their response during implantation, we have to be careful in the handling of thermogels in the clinical setting. There is a need to reduce or remove the antagonistic influence of thermogels on biological systems. This demands the use of naturally existing materials that can contribute in metabolism and elimination, or depend on starting materials that are benign to biological systems. This has to be monitored on different levels of biological systems; the molecular level where disturbances of innate bio-molecular assemblies need to be circumvented, the cellular level where antagonistic effects show due to physical changes in sub-cellular assemblies, tissue/organ levels where the special anatomical structures, such as the junctional arrangement of cells or tubular organization of blood vessels, should not be conceded. These deliberations are augmented especially when we generate designer structures or nano-structured materials. The best way to ensure safety is to rely on degradable thermogels that does not leave any trace of the thermogel behind after the thermogel completes its task in the body, and with degradation products that are either excreted directly or can enter into natural metabolic pathways. The safety analysis should be thorough; a corresponding probe of body fluids, immune, and other surveillance systems need to be analysed to guarantee thermogel compliance with the biological system in the short term. Given the possibility of altering the expression of a wide range of genes whenever a therapeutic agent is introduced into the body, long-term assessments for genotoxicity of the thermogel will be needed. Finally, we should expect any unexpected effects, but also be assured that development will be made by overcoming the presented problems. Preclinical models, designed for the intended use and characterized carefully, are the finest tools to follow in safety studies before the thermogels find their way to clinical utility.
